# The sonographic digital portfolio: a longitudinal ultrasound image tracking program

**DOI:** 10.1186/2036-7902-4-15

**Published:** 2012-07-02

**Authors:** Daralee R Hughes, Erika Kube, Brad D Gable, Francis E Madore, David P Bahner

**Affiliations:** 1 Department of Emergency Medicine, The Ohio State University Medical Center, 760 Prior Health Sciences Library, 376 W 10th Ave, Columbus, OH, 43210, USA; 2 Summa Health Systems, Akron, OH, 44309, USA; 3 Hennepin County Medical Center, Minneapolis, MN, 55415, USA; 4 Ohio Health System, Columbus, OH, 43215, USA

**Keywords:** Medical education, Digital portfolio, Ultrasound images

## Abstract

**Background:**

Ultrasonography (US) at the medical student level is developing. As clinical skills and simulation centers expand, US equipment miniaturizes, and more students are exposed to ultrasound; a digital portfolio comprised of US images and videos may be useful in demonstrating experience and possibly competency.

**Methods:**

Medical students participated in US curricula consisting of didactics and hands-on training. From 1 July 2006 to 30 June 2008, student images and videos were saved. Total images and videos were evaluated and catalogued.

**Results:**

A total of 10,074 images and 1,227 videos were saved during the 2-year period. For the academic year 2006 to 2007, 159 medical students obtained 3,641 of the images (84.9%) and 270 of the videos (86.0%). First year students obtained 778 images and 20 videos; second year students, 1,174 images and 64 videos; third year students, 211 images and 20 videos; and fourth year students, 1,478 images and 166 videos.

For the academic year 2007 to 2008, 222 medical students obtained 4,340 images (75%) and 619 videos (67.8%). First year students obtained 624 images and 109 videos; second year students, 555 images and 81 videos; third year students, 132 images and 14 videos; and fourth year students, 3,029 images and 415 videos.

**Conclusions:**

The ultrasound digital portfolio allows medical students to collate and document their ultrasound experience. Currently, there is no requirement for ultrasound training, documentation of competency, or minimum numbers of US exams for medical education. The ultrasound digital portfolio may be a useful tool in documenting ultrasound proficiency.

## Background

Ultrasonography is part of the curriculum of most emergency medicine (EM) residency programs; however, ultrasound education at the medical student level is still developing and not uniformly part of the educational curriculum [1-6]. Early exposure to education in ultrasonography has been advocated to improve proficiency, confidence, and competency, which could be beneficial prior to entering any residency. It has been shown, in a range of educational experiences, that medical students of various training levels can attain a level of proficiency in ultrasonographic technique and interpretation [7-10]. As clinical skills and simulation centers become more numerous and ultrasound equipment becomes more portable, educational and hands-on experience with ultrasound can be provided to all levels of medical trainees. As ultrasound experience expands, it becomes increasingly important for physicians to demonstrate mastery of certain skill sets. A digital portfolio comprised of ultrasonographic images and videos performed by an individual may be useful in demonstrating experience and competency.

## Methods

In July of 2006, The Ohio State University Medical Center acquired three Sonosite MicroMaxx (Sonosite, Inc., Bothell, WA, USA) ultrasound machines and eight transducers including curvilinear, linear array, endoluminal, and phased array for use in the Clinical Skills Education and Assessment Center (CSEAC), a clinical skills laboratory at The Ohio State University Medical School. Practical ultrasound exams were completed using volunteer models and practice phantoms. With the capacity to offer ultrasound scanning experience to a large number of students, medical students from all 4 years (Med 1 to 4) were able to participate in year-specific ultrasound curricula including both didactics and hands-on training (Table 1). First year students (Med 1) were able to sign up for an extracurricular ‘Ultrasound Academy’ which involved a combination of didactic sessions and hands-on scanning sessions taught by a combination of EM attending physicians/residents and fourth year (Med 4) ultrasound students. Med 1 students learned the basic principles of ultrasound, the various uses and indications for ultrasound, and the technical components of the machine. They also put their skills to use by learning and practicing the Focused Assessment with Sonography for Trauma (FAST) exam. Med 2 students could participate in the Model Pool as simulated patients for various ultrasound courses throughout the year. In addition, they participated in didactic and hands-on sessions in which they were taught more advanced ultrasound scans including echocardiography evaluation of the abdominal aorta, OB/GYN (transvaginal and transabdominal), hepatobiliary, thoracic, and fluid status assessment (using inferior vena cava (IVC) measurements). In addition, Med 2 students were given the opportunity to participate in clinical scanning shifts in the emergency department with the Ultrasound Director. Med 3 students who had been a part of either the first year Ultrasound Academy or the second year Model Pool were encouraged to continue practicing their sonography skills at their convenience by setting up individual or small group sessions in the CSEAC and saving these images to their portfolio. All Med 3 students received a basic introduction to ultrasound lecture with a hands-on session as part of their required Clinical Skills Immersion Experience curricula but are not required to save images during this session. Med 4 students could participate in a longitudinal year-long elective Honors Ultrasound course. Students in this course were required to attend monthly didactic sessions and journal club discussions focused on ultrasound topics as well as participate in one hands-on session in the CSEAC each month. Didactic sessions are taught by the Ultrasound Director and faculty from other medical specialties with a specific interest in ultrasound and who use ultrasound in their daily practice as well as senior sonographers. The didactic curriculum is structured to cover the major uses and indications for physician-performed ultrasound. They were also required to conduct an ultrasound research project for presentation. Journal club sessions are led by the Ultrasound Director with each student giving a brief overview of one article throughout the year. Med 4 students were encouraged to set up additional hands-on sessions in the CSEAC and schedule clinical scanning shifts in the emergency department with the Ultrasound Director.

**Table 1:** Curriculum content for each ultrasound program and resources used

**Program**	**Content**	**EM SONO modules**	**Online quizzes**	**Didactic sessions**^**a**^
*Med 1:* Trinity	1. Cardiac - SUX, LAX, SAX	1. FAST	One quiz for each EM SONO module	1. Basic US - overview of Trinity protocol and images
		2. Echocardiography		
	2. Aorta - SMA, bifurcation	3. Aorta		
	3. Posterior cul-de-sac			2. Midyear review of Trinity protocol
	4. Perihepatic			
	5. Perisplenic			
*Med 2:* Trained as simulated ultrasound patient	1. FAST	1. FAST	One quiz for each EM SONO module	1. FAST
	2. Cardiac	2. Echocardiography		2. Echocardiography
	3. Aorta - celiac trunk, SMA, bifurcation	3. Aorta		3. Aorta
		4. Critical care		4. Critical care
	4. Critical care - IVC, lung sliding	5. Vascular access		5. Vascular access and procedures
	5. Vascular access			
*Med 3:* CSIE	1. Vascular access (required)	1. Practical scanning	One quiz for each EM SONO module	Basic US and overview of each station
		2. Vascular access		
	2. Cardiac - LAX, SAX	3. FAST		
		4. Aorta		
	3. FAST	5. First trimester OB (all optional)		
	4. Aorta - SMA, bifurcation			
	5. Transvaginal US			
	6. Critical care - IVC			
*Med 4:* Honors US	1. FAST	1. Practical scanning	One quiz for each EM SONO module required	Didactic - 1 per month Journal club - 1 per month
	2. Echocardiography - LAX, SAX, A4C, SUX	2. FAST		
		3. Critical care		
		4. Aorta		
	3. Aorta - celiac trunk, SMA, bifurcation	5. First trimester OB		
			6. Renal		
	4. Critical care - IVC, lung sliding	7. Hepatobiliary			
	5. Pelvic	8. Vascular access			
	6. Hepatobiliary	9. Testicular torsion			
	7. Renal				
	8. Procedural guidance				
	9. Ophthalmology				
	10. Soft tissue, musculoskeletal				

^a^Didactic sessions - powerpoint presentations and handouts utilized. *CSIE*, Clinical Skills Immersion Experience; *US*, ultrasound; *SUX*, subxiphoid; *LAX*, parasternal long axis; *SAX*, parasternal short axis; *SMA*, superior mesenteric artery; *FAST*, Focused Assessment with Sonography for Trauma; *IVC*, inferior vena cava; *A4C*, apical four chamber; *OB*, obstetrics.

During the two full academic years from 1 July 2006 to 30 June 2008, all of the aforementioned courses were under way in addition to EM resident and various hospital department ultrasound-training sessions. The first 2 months of data could not be retrieved as the login procedures were being implemented. Additionally, many of the images/videos obtained during the Med 3 optional sessions in the CSEAC were not saved as this was an optional rather than a mandatory requirement. All images and videos that were obtained during the 22 months by students and faculty in the CSEAC were saved to Compact Flash disk and copied to a central computer server during that time period. At the end of the 2 years, the videos and images were compiled, sorted by training level (Med 1 to 4, postgraduate year, and attending) and were evaluated. For each scan, the participant's name, training level, number of still images and video clips, and type of scan(s) were catalogued. This study was determined to be exempt by the OSU Biomedical Institutional Review Board.

Pelvic ultrasound scans were completed using a Blue Phantom endovaginal ultrasound trainer (Blue Phantom, Kirkland, WA, USA) that simulates a first trimester pregnancy. Vascular scans were completed using linear array transducers for peripheral vessels and those in the neck, while a curvilinear array transducer was used for the abdominal aorta. Vessel cannulation was practiced using a Blue Phantom (Advanced Medical Technologies, Redmond, WA, USA) vein simulator.

## Results

A total of 10,074 images and 1,227 videos were saved during the 22 months. Of these images, medical students were responsible for 7,981 of the images (79.2%) and 889 of the videos (72.5%).

The academic year 2006 to 2007 was comprised of 4,291 images and 314 videos. A total of 159 medical students were responsible for 3,641 of the images (84.9%) and 270 of the videos (86%) (Table 2). Forty-eight first year medical students (Med 1) obtained 778 images and 20 videos, 68 second year medical students obtained (Med 2) 1,174 images and 64 videos, 20 third year medical students (Med 3) obtained 211 images and 20 videos, and 23 fourth year medical students (Med 4) obtained 1,478 images and 166 videos (Table 3).

**Table 2:** Number and percentage of images and videos by rank of participant for 2006 to 2007

**2006 to 2007**			
**Rank**	**Images**	**Videos**	**Number of participants**
Med 1 to 4	3,641 (84.9%)	270 (86%)	159 (70.4%)
PGY	147 (3.4%)	19 (6.1%)	26 (11.5%)
Attending	36 (0.84%)	5 (1.6%)	4 (1.8%)
Unknown rank	467 (10.9%)	20 (6.4%)	37 (16.4%)
Total	4,291	314	226

PGY, postgraduate year.

**Table 3:** Number and percentage of images and videos by student training level for 2006 to 2007

**2006 to 2007**			
**Medical student rank**	**Images**	**Videos**	**Number of participants**
Med 1	778 (21.4%)	20 (7.4%)	48 (30.2%)
Med 2	1,174 (32.2%)	64 (23.7%)	68 (42.8%)
Med 3	211 (5.8%)	20 (7.4%)	20 (12.6%)
Med 4	1,478 (40.6%)	166 (61.5%)	23 (14.5%)
Total	3,641	270	159

The remainder of the 5,783 images and 913 videos were obtained during the 2007 to 2008 academic year. Of these ultrasound scans, 222 medical students obtained 4,340 images (75%) and 619 videos (67.8%) (Table 4). Sixty-five first year medical students (Med 1) were responsible for 624 images and 109 videos, 45 second year students (Med 2) obtained 555 images and 81 videos, 28 third year students (Med 3) obtained 132 images and 14 videos, and 84 fourth year students (Med 4) obtained 3,029 images and 415 videos (Table 5).

**Table 4:** Number and percentage of images and videos by rank of participant for 2007 to 2008

**2007 to 2008**			
**Rank**	**Images**	**Videos**	**Number of participants**
Med 1 to 4	4,340 (75%)	619 (67.8%)	222 (63%)
PGY	812 (14%)	158 (17.3%)	54 (15.3%)
Attending	371 (6.4%)	84 (9.2%)	22 (6.3%)
Unknown rank	260 (4.5%)	52 (5.7%)	54 (15.3%)
Total	5,783	913	352

PGY, postgraduate year.

**Table 5:** Number and percentage of images and videos by student training level for 2007 to 2008

**2007 to 2008**			
**Medical student rank**	**Images**	**Videos**	**Number of participants**
Med 1	624 (14.4%)	109 (17.6%)	65 (29.3%)
Med 2	555 (12.8%)	81 (13.1%)	45 (20.3%)
Med 3	132 (3.0%)	14 (2.3%)	28 (12.6%)
Med 4	3,029 (69.8%)	415 (67%)	84 (37.8%)
Total	4,340	619	222

## Discussion

Ultrasound training in medical school is not ubiquitous despite references of ultrasound being the stethoscope of the twenty-first century. The technology has outpaced the current education, and this ultrasound program was developed to provide an experience and exposure to ultrasound during medical school. The medical student ultrasound digital portfolio is a means for students to collate their ultrasound experience and have documentation of their exams in a digital format as proof of ultrasound experience for residency. Graded ultrasound exposure for medical students allows repeated opportunities to become comfortable with the ultrasound equipment and the protocols for scanning patients. Students are given direct feedback and suggestions for improvement by the proctors and facilitators at each hands-on session. Students were also assessed by a combination of online quizzes (optional and required depending on the particular program), written exams, and practical hands-on exams which allow for a more standardized evaluation of the students' knowledge and provide a gauge for each student to monitor their progress. The primary proctors for the various ultrasound programs are directly trained by the Ultrasound Director. Med 4 students in the Honors Ultrasound course have completed 3 years of ultrasound training under the mentorship of the Ultrasound Director and work with the EM residents who also receive one-on-one teaching and instruction from the Ultrasound Director as well as utilize the same online resources for educational enhancement. This creates uniformity and consistency in the educational methods used for instruction. By each instructor teaching consistent ultrasound techniques throughout the various ultrasound programs, the repetition provides for more efficient teaching.

The future requirements for medical students in regard to specialty-specific scope of practice guidelines will have to be explored by national organizations. Currently, there is no formal requirement for ultrasound training or for documentation of competency. Future practice may codify the ultrasound requirements that are expected before the start of residency and during residency of specialties that use ultrasound in their practice. The above method was used as a way to centralize ultrasound resources and provide opportunities to multiple students at various stages of medical education to prepare them for ultrasound use in clinical practice. It also provided a progressive curriculum for those interested and committed to learning ultrasound during the early stages of their medical education.

Several questions will need to be addressed as ultrasound education advances in the future. Should medical students be taught ultrasound? If so, where in the current curriculum is it best suited? Should students be responsible managing their own digital portfolio with a jump drive? Should all image exams be stored on a hospital radiology server (such as a PACS system)? Recent advances in technology and the availability of web-based data storage systems (clouds) have provided an additional option for image storage. Students would be able to access their images, and instructors could track the number and types of images each student has completed as well as provide feedback on the quality of images for students to review. How should residency programs interface with medical students for the types and amount of training that can be recommended before residency begins? Is this ultrasound skill set a continuum over medical school and residency? The answers to these questions will need to be refined in medical education forums across the country with specialty organizations, residency programs, and medical schools collaborating on requirements.

### Limitations

As no formal criteria exist for focused assessment of most body parts, students were given credit for a ‘scan’ of a particular organ if they had logged at least one image of that organ. FAST exams required at least one of the four views. If the student captured a single view of the heart, kidney, liver, spleen, or bladder, they were given credit for a FAST exam. If they captured two or more different views of any organ, they were given credit for an organ-specific exam (i.e., ‘cardiac’ or ‘hepatobiliary’). Students were given credit for the pelvic (i.e., gynecological) and obstetric (i.e., fetal) portions of the exam as two separate ‘scans.’

There was no standardized login nomenclature established for those practicing ultrasound in the CSEAC, which led to difficulty when collating and analyzing the scans due to the differences in information that each person entered upon logging onto an ultrasound machine. Retrospectively cataloguing scans required determination of where each participant was in their medical training at the time the scan was performed which unfortunately led to a large number of ‘unknown rank’ classifications in the data. In addition, there were some individuals that could not be accurately identified by name so were also given the ‘unknown rank’ designation. A standardized login nomenclature, which includes the student's name and year in training, has now been developed for all participants to ensure consistency throughout the ultrasound programs and assist in cataloguing, data collection, and collating images for each participant's digital portfolio.

Med 3 students were not required to save images during the Clinical Skills Immersion Experience which further limited the completeness of the data as well as being able to accurately assess how students are progressing in their ultrasound training. More formalized requirements for each year of ultrasound training, in addition to improved nomenclature and storage procedures, will allow for more accurate determination of ultrasound utilization by each student and the progression of knowledge acquisition.

## Conclusions

In many specialties, portable ultrasound continues to improve and enhances patient care. Early education of operators is a priority that can begin to be addressed in medical school. The practice of ultrasound has clearly been shown to be operator-dependent, and the way to train better operators is to start early, provide opportunities for practice, and standardize curriculum that will ultimately align with residency requirements in the various specialties.

## Endnotes

An abstract utilizing some of the data in this report was presented in poster form at the American Institute of Ultrasound in Medicine Conference in March 2010.

## Competing interests

The authors declare that they have no competing interests.

## Authors’ contributions

DRH participated in the acquisition and analysis of the data as well as in the drafting and revision of the manuscript for submission. EK participated in the conception and design of the study and in the revision of the manuscript. BDG participated in the conception, design, and coordination of the study as well as in the acquisition and analysis of the data and helped draft the manuscript. FEM contributed to the acquisition and analysis of the data and helped draft and revise the manuscript. DPB conceived the study and participated in the design and coordination as well as in the revision of the manuscript. All authors read and approved the final manuscript.

## Authors’ information

DRH is a second year Emergency Medicine resident at The Ohio State University, Columbus, OH, USA. EK is an Emergency Medicine physician at Ohio Health Systems, Columbus, OH, USA. BDG is a third year Emergency Medicine resident at Summa Health Systems, Akron, OH, USA. FEM is a third year Emergency Medicine resident at Hennepin County Medical Center, Minneapolis, MN, USA. DPB is an associate professor at the Department of Emergency Medicine and is the Director of Ultrasound at The Ohio State University Medical Center, Columbus, OH, USA.
